# Effect of Withdrawal Rate on Solidification Microstructures of DD9 Single Crystal Turbine Blade

**DOI:** 10.3390/ma16093409

**Published:** 2023-04-27

**Authors:** Yanpeng Xue, Xiaoguang Wang, Jinqian Zhao, Zhenxue Shi, Shizhong Liu, Jiarong Li

**Affiliations:** Science and Technology on Advanced High Temperature Structural Materials Laboratory, AECC Beijing Institute of Aeronautical Materials, Beijing 100095, China

**Keywords:** DD9 single crystal superalloy, directional solidification, withdrawal rate, dendrite morphology, γ′ precipitation, γ–γ′ eutectics

## Abstract

Single crystal superalloys are widely used in the manufacturing of turbine blades for aero-engines due to their superior performance at high temperatures. The directional solidification process is a key technology for producing single crystal turbine blades with excellent properties. In the directional solidification process, withdrawal rate is one of the critical parameters for microstructure formation and will ultimately determine the blade’s properties. In this paper, the as-cast microstructures in the typical sections of a DD9 single crystal (SX) superalloy turbine blade were investigated with 3 mm/min and 5 mm/min withdrawal rates during the directional solidification process. With increased withdrawal rate, the dendrite morphologies tended to become more refined, and the secondary dendritic arms tended to be highly developed. The dendrite in the blade aerofoil section was more refined than that in the tenon section, given the same withdrawal rate. Additionally, with increasing withdrawal rates, the size and dispersity of the γ′ precipitates in the inter-dendritic (ID) regions and dendritic core (DC) tended to decrease; furthermore, the size distributions of the γ′ precipitates followed a normal distribution law. Compared with the ID regions, an almost 62% reduction in the average γ′ sizes was measured in the DC. Meanwhile, given the same withdrawal rate, at the blade’s leading edge closest to the heater, the γ′ sizes in the aerofoil section (AS) were more refined than those in the tenon section (TS). As compared with the decreasing cross-sectional areas, the increased withdrawal rates clearly brought down the γ′ sizes. The sizes of the γ–γ′ eutectics decreased with increasing withdrawal rates, with the γ–γ′ eutectics showing both lamellar and rosette shapes.

## 1. Introduction

Single crystal (SX) superalloys are widely used as blades and vanes in the hot sections of aero-engine gas turbines due to their excellent high-temperature properties [1,2,3]. In order to satisfy the ever-increasing turbine entry temperature (TET) demands required for enhancing the efficiency of engines and turbines, turbine blades and vanes need to work in high temperatures, ones very close to the melting point of the SX superalloys they are made of [4]. Compared to second generation SX superalloys, third generation SX superalloys have higher temperature capabilities, making them attractive for use in advanced gas turbine engines [1,5]. DD9 alloy is a low-cost third generation SX superalloy developed with independent intellectual property rights in China [1]. Compared with the DD6 superalloy, which is widely used in the hot end parts of aero-engine gas turbines [2,3], DD9 alloy has an approximately 30 °C improvement in temperature capability. Thus, many studies on the microstructure and properties of DD9 SX superalloy have been carried out by many researchers [6,7,8,9,10].

It is well known that directional solidification technology has played a crucial role in the development of single crystal blades and vanes [11,12]. In the directional solidification process, solidification parameters such as withdrawal rate, temperature gradient and alloy composition can prompt solidification microstructures of SX blades and vanes to align in [001] orientation, which reveals their excellent high-temperature performance [13,14,15,16,17,18,19]. Mirak et al. [20] suggested that an increase in the withdrawal rate reduced the fraction of porosity and eutectic phase and increased the γ′ particles, which resulted in an improvement in the hot tensile strength. Lu et al. [21] summarized their argument by saying that the addition of Re could enhance the creep property under each stress, which contributed an essential insight for optimizing the alloying design and enhancing long-term reliable service of industrial gas turbine blades. Chen et al. [22] reported that the addition of 0.5 wt.% Ti resulted in more and finer γ′ precipitates, narrower γ channels, and a higher magnitude of the γ/γ′ lattice misfit. The creep stain rate decreased significantly, and the creep life extended to twice that of the base alloy at 1030 °C and 230 MPa. Wang et al. in [23] illustrated that a thermal gradient which was 10–12 times larger than that of the Bridgman process could be achieved by using the DWDS process. As compared to the Bridgman process, a more refined microstructure was produced, one having significantly more refined dendritic structures, finer γ′ precipitates and smaller and more homogeneously distributed γ/γ′ eutectic pools in the DWDS samples. Szeliga et al. [24] indicated that refinement of the dendritic microstructure in the CMSX-4 Ni-based single crystal casting blade was manufactured using standard adjusted or novel inner radiation baffles, which increased thermal gradients in the industrial-scale Bridgman solidification process.

Fortunately, the above solidification parameters could be controlled independently during directional solidification [13]. In the directional solidification process, the withdrawal rate is one of the important solidification parameters, one which affects the temperature gradient and the dendrite growth rate at the solid–liquid interface and mainly determines the microstructure of the SX blade [25,26,27]. Wang et al. [28] stated that, with increased withdrawal rates, the average primary and secondary dendrite arm spacings decreased, the average sizes of the γ′ precipitates and the γ/γ′ eutectic of a nickel-based superalloy were reduced, and the chemical segregations were alleviated. Zhao et al. [29] reported that the segregation of elements was suppressed and the size of the γ′ phase decreased significantly in an AM3 nickel-based superalloy with increasing withdrawal rates. Liu et al. [30] studied the effect of solidification conditions on micropores formed during homogenization of a Ni-base SX superalloy. It was reported that primary dendrite arm spacing increased with increases in the withdrawal rate. Ai et al. [31] investigated the solidification behaviours of a Mo-rich Ni3Al-based SX superalloy at different withdrawal rates. It was concluded that, because of solid back diffusion during solidification, the volume fraction of inter-dendritic precipitation initially increased and then decreased with an increased withdrawal rate. Liu et al. [32] found that the size and volume fraction of the eutectics of a nickel-base SX superalloy decreased with increases in withdrawal rate during directional solidification. Zhang et al. [33] reported that the primary and secondary dendrite arm spacing of a SX Ni-base superalloy decreased with an increasing withdrawal rate. Lian et al. [34] establishes that an increased withdrawal rate reduced the dendritic arm spacing, refined the dendritic structures, aggravated element (e.g., W, Al, Ta, Ti, etc.) segregation, and reduced the size and content of the spherical γ′ phase in the dendrite core and the inter-dendritic region. Moreira et al. [35] observed that the dendritic microstructure presented a primary dendrite arm spacing around 370 µm (for a withdrawal rate of 3.33 mm/min) and 320 µm (for 5.0 mm/min).

However, in these studies, the effects of withdrawal rate on microstructural morphologies of SX nickel-based superalloys were based on simplified geometrical castings, such as rods and blade models [28,29,30,31,32,33,34,35,36]. Compared with simplified geometrical castings, the structures of hollow blades for aero-engines are more complex, which leads to varieties of radiation heat transfers in different positions of the turbine blades during the directional solidification process. The solidification microstructures of turbine blades will be affected by radiation heat transfers. Therefore, in order to optimize the solidification process parameters of DD9 SX blade in industry and improve the performance of aero-engines, the effects of withdrawal rates on the solidification microstructures of a DD9 SX superalloy turbine blade were investigated in this paper.

## 2. Experimental

**Figure 1:**
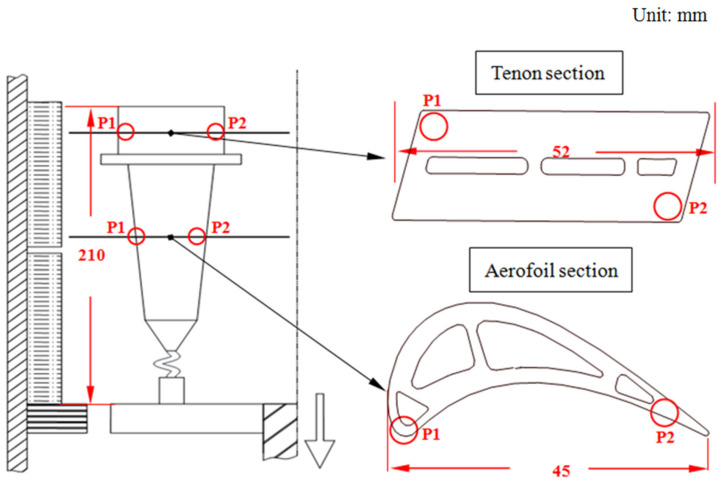
Diagram of two sectional positions of the SX superalloy turbine blade in a directional solidification furnace (P1: leading edge; P2: trailing edge).

A low-cost third generation SX Ni-based superalloy DD9 with the chemical composition described below was employed in this work, as shown in Table 1 [1]. The DD9 SX blade was directionally solidified by the spiral grain selector method in a vacuum-induction directional-solidification furnace with a high temperature gradient. The position of the typical section of the SX blade is shown in Figure 1. The DD9 master alloy was overheated to 1580 °C in a crucible poured into the two sets of ceramic mould shells at 1525 °C and put in contact with the chilling plate. When a stable temperature gradient was established at the solid–liquid interface, in order to maintain the continuity of the SX growth, two sets of ceramic mould shells full of melt were removed from the heating zone to the cooling zone of the furnace with the withdrawal rates of 3 mm/min and 5 mm/min, respectively. The withdrawal rate between 3 mm/min and 5 mm/min has commonly been used in the directional solidification process of turbine blade industrial production, as well as previous studies [20,26,27,29,31,32,33,34,35,36,37]. When the withdrawal rate was lower than 3 mm/min, it affected production efficiency of turbine blades, but when it was higher than 5 mm/min, the height of the blade’s ‘mushy zone’ was increased, and the tendency towards crystal defect formation, such as sliver grains and stray grains [26], was promoted. In addition, in order to compare the solidification mechanism with that of other projects in the literature, the withdrawal rates of 3 mm/min and 5 mm/min were considered optimal. The misorientation between the [001] growth direction and the principal stress axis of the DD9 SX blade was less than 10°.

The cross-section specimens of the DD9 SX blade parallel to the crystallographic plane (001) were taken from typical sectional positions, and polished for microstructural analysis. The polished samples were etched by a chemical etching solution of 100 mL H_2_O + 80 mL HCl + 25 g CuSO_4_ + 5 mL H_2_SO_4_. The observation sectional positions of the specimens were marked, defined as the circle area of P1 and P2 in Figure 1. The dendrite morphologies of the specimens were observed by an optical microscope (OM), and the microstructures of the γ′ precipitates and the γ–γ′ eutectics were observed through a scanning electron microscope (SEM). The various γ′ size distributions of the specimens were analysed by using metallurgical analysis software (Image Pro Plus version 6.0). Energy-dispersive X-ray analysis (EDX) was implemented to evaluate the element’s distribution within the dendritic structure.

## 3. Results and Discussion

### 3.1. Dendrite Morphologies

**Figure 2:**
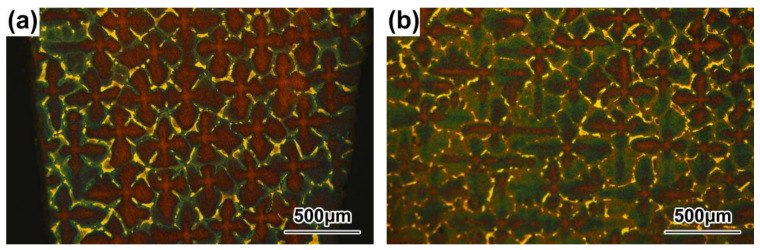
Dendrite morphologies of DD9 blade aerofoil sectional P2 positions with two withdrawing rates: (**a**) 3 mm/min and (**b**) 5 mm/min.

**Figure 3:**
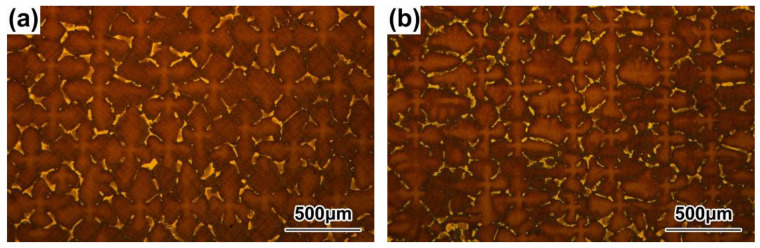
Dendrite morphologies of DD9 blade tenon sectional P2 positions with two withdrawing rates: (**a**) 3 mm/min; (**b**) 5 mm/min.

The dendrite morphologies in the P2 positions of the DD9 SX superalloy blade aerofoil and tenon sections with the withdrawal rates of 3 mm/min and 5 mm/min, are shown in Figure 2 and Figure 3, respectively. With the increase of the withdrawal rate, the dendrite morphologies of the DD9 blade aerofoil and the tenon sectional P2 positions tended to become more refined; in addition, the secondary dendrite arms tended to be highly developed and the tertiary dendrites only partly appeared, which was in agreement with the results of Szeliga et al. [36] and Gancarczyk et al. [37]. Furthermore, within the same withdrawal rate, the primary dendrite arm spacing (PDAS) in the TS was larger than that of the AS listed in Table 2. The above finding was consistent with recent research, which has suggested that the primary dendrite arm spacing increases with increasing wall thickness [36,38]. The dendrite morphologies were related to temperature gradient G and dendrite growth rate V at the solid–liquid interface during the growth of dendrite. The primary dendrite arm spacing (PDAS) and the secondary dendrite arm spacing (SDAS) were determined by the dendritic growth laws G^−1/2^V^−1/4^ and G^−1/3^V^−1/3^ [39], respectively. Thus, at the same cross-sectional area, when the temperature gradient G was constant at the solid–liquid interface, the higher the withdrawal rate V was, the smaller the PDAS was; meanwhile, the cooling rate at the solid–liquid interface was faster, and the transverse temperature gradient was higher, which induced the secondary dendrite arms to be more developed. Furthermore, the PDAS depended on the heat dissipation at the solidification interface within the same withdrawal rate. Radiation was the main mode of heat transfer in a vacuum induction melting furnace during the dendrite growth process of the SX blade; the cross-sectional area in the TS was larger than that in the AS of the blade, which led to slow radiation heat transfer in the TS. Therefore, the longitudinal temperature gradient of the TS was smaller than that of the AS, resulting in a larger PDAS in the TS.

### 3.2. Sizes and Morphologies of the γ′ Precipitates

**Figure 4:**
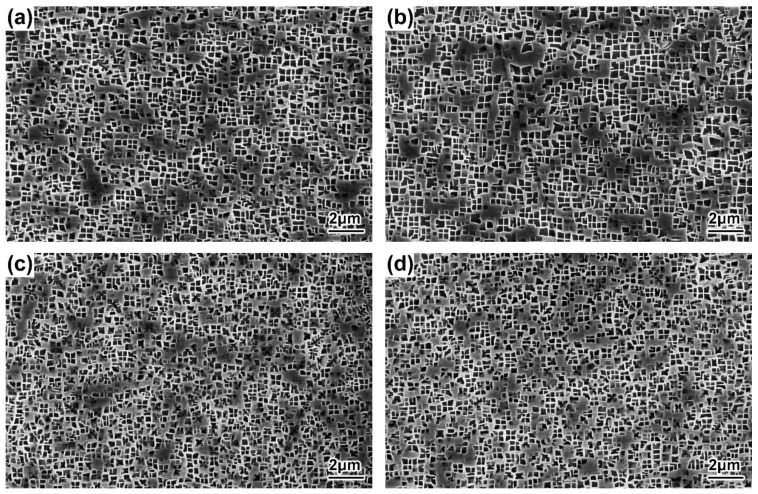
Microstructures of the γ′ precipitates in the DC of the DD9 SX blade aerofoil, sectional P1 and P2 positions, with two withdrawing rates: (**a**) 3 mm/min, P1; (**b**) 3 mm/min, P2; (**c**) 5 mm/min, P1; and (**d**) 5 mm/min, P2.

**Figure 5:**
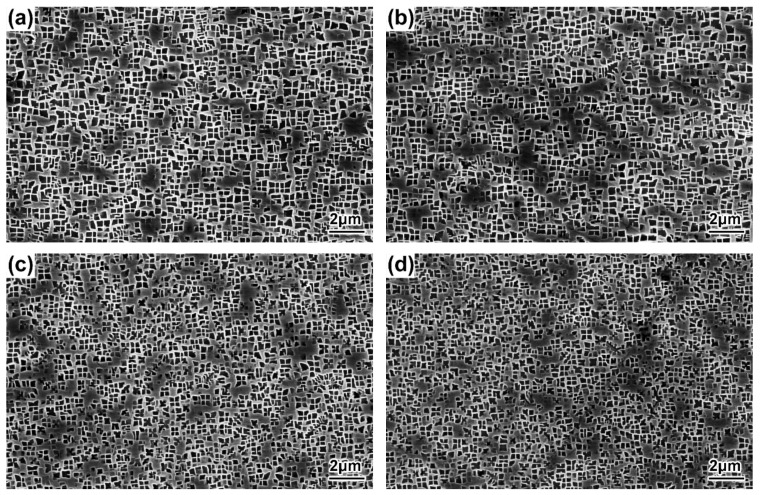
Microstructures of the γ′ precipitates in the DC of the DD9 SX blade tenon, sectional P1 and P2 positions, with two withdrawing rates: (**a**) 3 mm/min, P1; (**b**) 3 mm/min, P2; (**c**) 5 mm/min, P1; and (**d**) 5 mm/min, P2.

**Figure 6:**
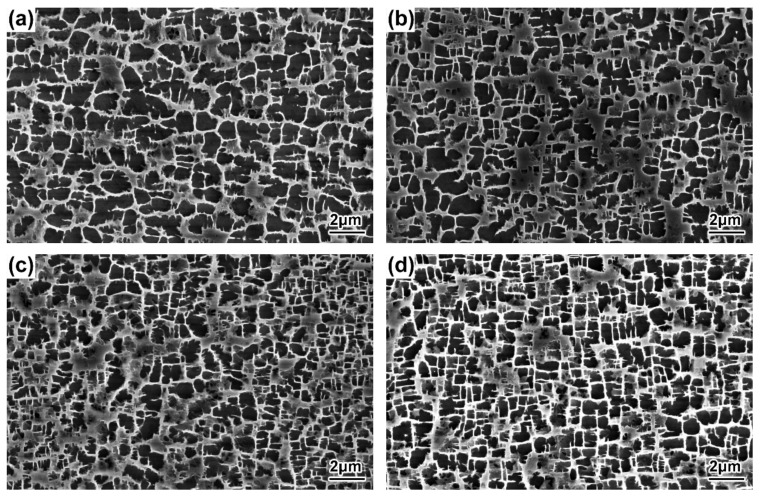
Microstructures of the γ′ precipitates in the ID regions of the DD9 SX blade aerofoil, sectional P1 and P2 positions, with two withdrawing rates: (**a**) 3 mm/min, P1; (**b**) 3 mm/min, P2; (**c**) 5 mm/min, P1; and (**d**) 5 mm/min, P2.

**Figure 7:**
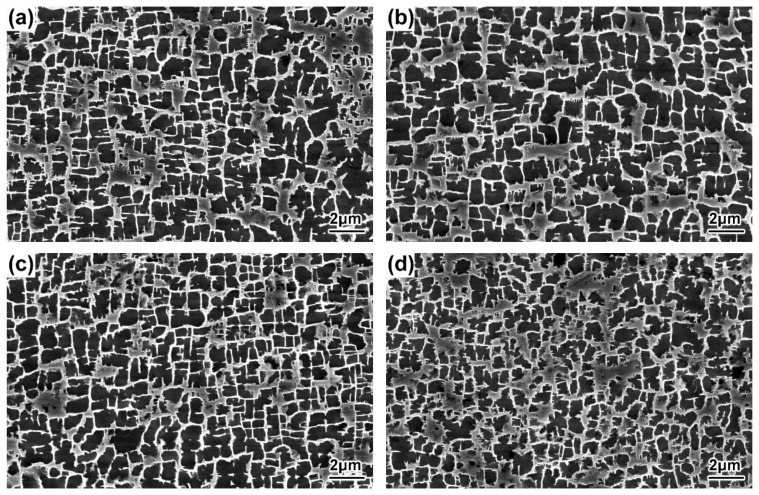
Microstructures of the γ′ precipitates in the ID regions of the DD9 SX blade tenon, sectional P1 and P2 positions, with two withdrawing rates: (**a**) 3 mm/min, P1; (**b**) 3 mm/min, P2; (**c**) 5 mm/min, P1; and (**d**) 5 mm/min, P2.

**Figure 8:**
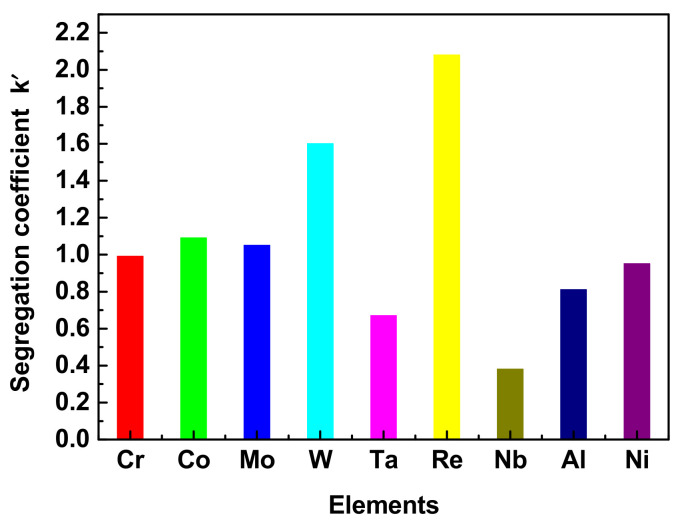
Segregation behaviour of the elements in the DC and ID regions of the DD9 SX superalloy.

The morphologies of the γ′ precipitates in the DC and ID regions of the DD9 blades’ typical sectional positions with two withdrawing rates are illustrated in Figure 4, Figure 5, Figure 6 and Figure 7. It can be seen that the sizes of the γ′ precipitates evinced a significant difference between the DC and ID regions in one dendrite area. Table 3 lists the average sizes of the γ′ precipitates in the DC and ID regions of the DD9 blades’ typical sectional positions with two withdrawing rates; it can be seen that the average sizes of the γ′ precipitates in the DC and ID regions were 0.278 μm and 0.737 μm, respectively. Thus, the average sizes of the γ′ precipitates in the DC were 62% less than those in the ID regions. In fact, the multicomponent segregations between the DC and ID regions played an important role in the differences of the γ′ sizes [37,40]. Because of the solute redistribution during the dendritic growth in the directional solidification process, some negative segregation elements of Co, W, Re would be segregated into the DC, and other positive elements, including Al and Ta, called γ′ forming elements [37], tended to be enriched in the last residual liquid, which solidified as the ID regions [29,41]. The element segregation behaviour of DD9 SX superalloy was given in Figure 8 [8].

The results shown in Figure 4, Figure 5, Figure 6 and Figure 7 also indicate that the average sizes of the γ′ precipitates in the same dendritic region at the same sectional positions of DD9 SX blade with a 5 mm/min withdrawal rate were smaller than those with a 3 mm/min withdrawal rate. Previous research efforts had reached the same result: that the increase in the withdrawal rate led to a decrease in the size of the γ′ precipitates [28,29]. This suggested that the variation of the γ′ precipitates’ size given different withdrawal rates was closely related to the cooling rate. There were two ways to form the γ′ precipitates from the supersaturated γ solid solutions in the DC and ID regions during the solidification of DD9 SX superalloy, namely γ → γ_DC_ + (γ′_ID_/γ_ID_) and γ_DC_ → γ′_DC_/γ_DC,_ respectively. In the nucleation stage of γ′ precipitation, with a 5 mm/min withdrawal rate, a higher cooling rate made the temperature drop below the γ′ solvus rapidly, which increased the supercooling degree of the nucleation of the γ′ precipitates [28,42,43]. The high supersaturation of the γ matrix then reduced the critical nucleation radius of the γ′ precipitates, and thus the γ matrix was rich in the nucleation cores of the γ′ precipitates. Within the process of withdrawing, a large number of nucleation cores of the γ′ precipitates entered the growth stage. The growth process of the γ′ precipitates mainly depended on the diffusion rates of the alloying elements [44], especially γ′ forming elements [28]. The growth of the γ′ precipitates was limited by the low diffusion rate, which was induced by insufficient thermal activation energy to drive the migration of γ′ forming elements from the γ matrix to the adjacent γ′ precipitates. Therefore, the γ′ precipitates were more refined within the 5 mm/min withdrawal rate.

**Figure 9:**
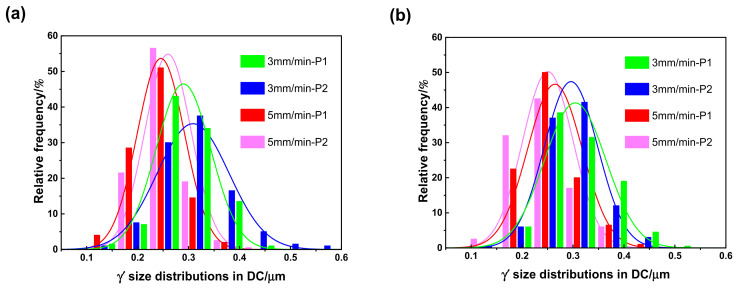
Size distributions of the γ′ precipitates in the DC of a DD9 SX blade aerofoil (**a**) and tenon (**b**), sectional P1 and P2 positions, with two withdrawing rates.

**Figure 10:**
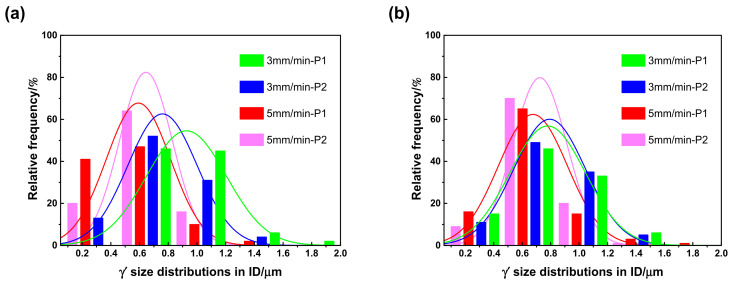
Size distributions of the γ′ precipitates in the ID regions of a DD9 SX blade aerofoil (**a**) and tenon (**b**), sectional P1 and P2 positions, with two withdrawing rates.

With the growth of the γ′ precipitates, the remaining supersaturation of the γ matrix was almost exhausted, while the coarsening process of the γ′ precipitates started to dominate the size distributions of the γ′ precipitates. In the solidification process, with a relatively lower cooling rate, some larger size γ′ particles had enough time to coarsen by combining with the neighbouring smaller γ′ particles, thereby reducing the interface energy around the smaller γ′ particles, while those large size γ′ particles away from the fine γ′ particles did not have the kinetic conditions to complete the coarsening process and maintained their original sizes. On the contrary, in the solidification process of a relatively higher cooling rate, there was little time for all the γ′ particles to complete the coarsening process. Thus, with the increase of the withdrawal rate, the cooling rate increased, the size dispersibility of the γ′ particles decreased, and all the size distributions of the γ′ particles followed a normal distribution law, as shown in Figure 9 and Figure 10.

**Figure 11:**
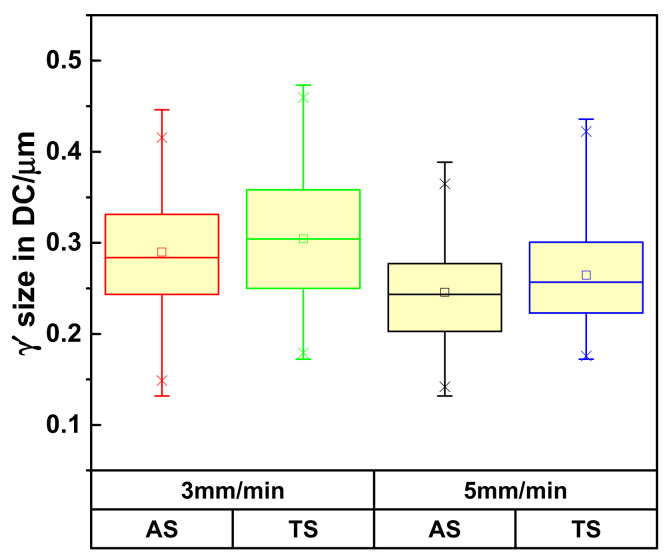
The γ′ sizes in the DC of DD9 SX blade aerofoil and tenon, sectional P1 positions, with two withdrawal rates.

In addition, given the same withdrawal rate, compared with the tenon sectional P1 positions, the γ′ sizes in the DC of DD9 SX blade aerofoil sectional P1 positions were more refined, as shown in Figure 11. The difference in the γ′ sizes in the aerofoil and tenon sectional positions of DD9 SX blade was closely related to the cross-sectional area. A comparison of the cross-sectional areas between the aerofoil and tenon is shown in Figure 1, and the calculation results show that the ratio of the cross-sectional areas between the tenon and the aerofoil of the blade was 4.6, with a tenon cross-sectional area of 830 mm^2^ and an aerofoil cross-sectional area of 180 mm^2^. Owing to the smaller cross-sectional area of the aerofoil, the transverse radiation heat dissipation capacity of the aerofoil section was stronger, which induced a large supercooling degree, a high nucleation rate and a short growth time of the γ′ precipitates, and finally resulting in a large number of γ’ precipitates with small sizes.

Although a large reduction in the cross-sectional area would result in smaller sizes of the γ′ precipitates, at least compared with the effect of the withdrawal rate on the sizes of the γ′ precipitates, this correlation was not significant, as shown in Table 4 and Table 5. This is because the increasing withdrawal rate directly caused the enhanced cooling rate in the longitudinal direction. However, even though the reduction of the cross-section area enhanced the lateral cooling capacity of the DD9 SX blade, the lack of the thermal convection in the furnace limited the increase of the cooling rate in the longitudinal direction.

### 3.3. Sizes and Morphologies of the γ-γ′ Eutectics

**Figure 12:**
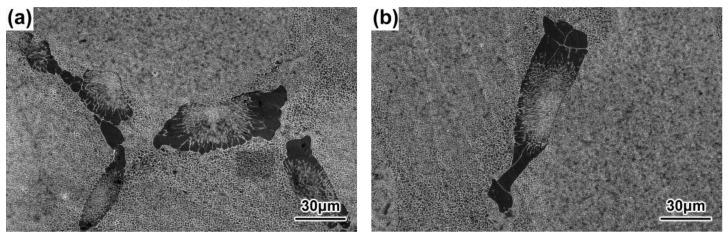
γ–γ′ eutectics of the DD9 SX blade aerofoil, sectional P1 (**a**) and P2 (**b**) positions, with a 3 mm/min withdrawal rate.

**Figure 13:**
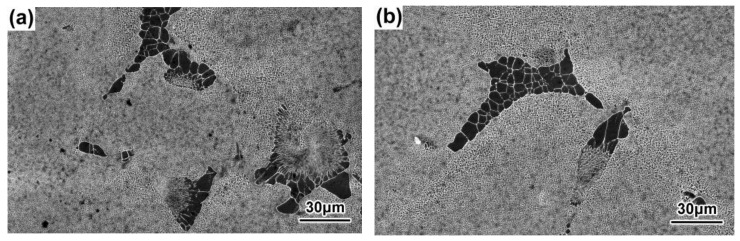
γ–γ′ eutectics of the DD9 SX blade aerofoil sectional, P1 (**a**) and P2 (**b**) positions, with a 5 mm/min withdrawal rate.

**Figure 14:**
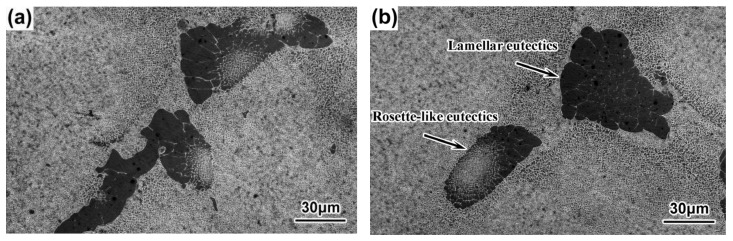
γ–γ′ eutectics of the DD9 SX blade tenon, sectional P1 (**a**) and P2 (**b**) positions, with a 3 mm/min withdrawal rate.

**Figure 15:**
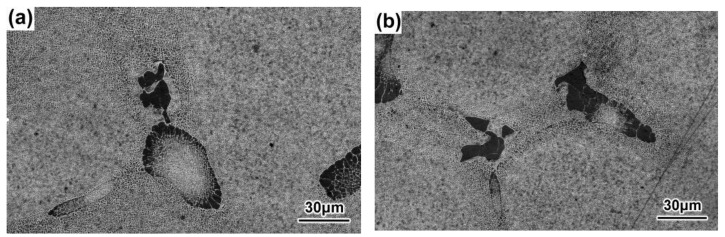
γ–γ′ eutectics of the tDD9 SX blade tenon, sectional P1 (**a**) and P2 (**b**) positions with a 5 mm/min withdrawal rate.

At the same cross-sectional position, the sizes of the γ–γ′ eutectics decreased, while the withdrawal rate increased, and the morphologies of the γ–γ′ eutectics showed both lamellar and rosette shapes, as shown in Figure 12, Figure 13, Figure 14 and Figure 15. Previous research has similarly suggested that the average size of the γ–γ′ eutectics decreased with an increase in the withdrawal rate [28,45]. When the SX superalloy solidified with a dendritic growth, the dendrites discharged solutes to the front in the ‘mushy zone’ at the solid–liquid interface. Meanwhile, the γ–γ′ eutectic structure formed between the dendrites when the positive segregating elements such as Al and Ta in the residual liquid reached eutectic composition. Therefore, the sizes of the γ–γ′ eutectics mainly depended on the segregation degree of composition in the final solidification zone of the SX superalloy. The transverse heat radiation increased with the increased withdrawal rate, the primary dendrites became refined, and the secondary dendrite arms were developed, which suppressed the degree of the inter-dendritic composition segregation. At the last, the diminution of residual liquid with the eutectic composition in the final solidification zone between the dendrites reduced the γ–γ′ eutectic growth space and induced the finer γ–γ′ eutectic structure.

## 4. Conclusions

In this paper, the effect of withdrawal rate on solidification microstructures of the DD9 SX turbine blade was studied. The research findings are as follows:

1. With the increase of withdrawal rates, the dendrite morphologies tended to become more refined, and the secondary dendritic arms tended to be highly developed. Additionally, the dendrite in the blade aerofoil section was more refined than that in the tenon section, given the same withdrawal rate.
2. The size and dispersity of the γ′ precipitates in the inter-dendritic regions and the dendritic core tended to decrease with increasing withdrawal rates. Moreover, the size distributions of the γ′ precipitates followed a normal distribution law. Compared with the inter-dendritic regions, the dendritic core exhibited a 62% reduction in the average γ′ size. Meanwhile, within the same withdrawal rate, the γ′ sizes in the aerofoil section were more refined than those in the tenon section. The increasing withdrawal rates resulted in a significant decrease in the γ′ sizes compared to the decreasing cross-sectional areas.
3. The sizes of the γ–γ′ eutectics decreased with increasing withdrawal rates, and the morphologies of the γ–γ′ eutectics exhibited both lamellar and rosette shapes.

## Figures and Tables

**Table 1:** Nominal composition for DD9 SX superalloy (wt/%) [1].

Cr	Co	Mo	W	Ta	Re	Nb	Al	Hf	C	Y	Ni
3.5	7	2	6.5	7.5	4.5	0.5	5.6	0.1	0.008	0.001	Bal

**Table 2:** PDAS of blades’ typical sectional positions with two withdrawing rates.

Withdrawal Rate (mm/min)	Section	PDAS/mm
3	AS	0.385
5	AS	0.377
3	TS	0.411
5	TS	0.405

**Table 3:** The average sizes of γ′ in the DC and ID regions of the DD9 SX blade, typical sectional positions, with two withdrawing rates.

Withdrawal Rate (mm/min)	Section	Position	d_mean_ (μm)	
			DC	ID Regions
3	AS	P1	0.289	0.927
3	AS	P2	0.309	0.760
5	AS	P1	0.248	0.592
5	AS	P2	0.259	0.645
3	TS	P1	0.304	0.779
3	TS	P2	0.296	0.793
5	TS	P1	0.264	0.675
5	TS	P2	0.251	0.725

**Table 4:** Withdrawal rate changed vs. γ′ size changed in the DC of the aerofoil and tenon, sectional P1 positions.

Withdrawal Rate Changed (mm/min)	Section	γ′ Size Changed (μm)
3→5	AS	0.289→0.248
	TS	0.304→0.264

**Table 5:** Cross-sectional area changed vs. γ′ size changed in the DC of the P1 positions with the same withdrawal rate.

Cross-Sectional Area Changed (mm^2^)	Withdrawal Rate (mm/min)	γ′ Size Changed (μm)
TS: 830→AS: 180	3	0.304→0.289
	5	0.264→0.248

## Data Availability

Not applicable.
